# Long-term Observational Study of the Longevity, Clinical Quality, and Periodontal Health Impact of Fiber-reinforced Composite Fixed Dental Prostheses

**DOI:** 10.3290/j.jad.c_2281

**Published:** 2025-09-25

**Authors:** Svenja Koeppe, Sebastian Soliman, Gabriel Krastl, Ralf Krug, Britta Hahn

**Affiliations:** a Svenja Koeppe* Dentist, Department of Conservative Dentistry and Periodontology, Center of Dental Traumatology, University Hospital Würzburg, Würzburg, Germany. Authored the manuscript; performed statistics and clinical examination for a doctoral degree.; b Sebastian Soliman* Dentist, Department of Conservative Dentistry and Periodontology, Center of Dental Traumatology, University Hospital Würzburg, Würzburg, Germany. Authored the manuscript, assessed clinical quality and periodontal health, was the principal investigator, and developed the idea. *both authors contributed equally to the article; c Gabriel Krastl Dentist, Department of Conservative Dentistry and Periodontology, Center of Dental Traumatology, University Hospital Würzburg, Würzburg, Germany. Proofread the manuscript, provided statistical advice.; d Ralf Krug** Dentist, Department of Conservative Dentistry and Periodontology, Center of Dental Traumatology, University Hospital Würzburg, Würzburg, Germany. Proofread the manuscript, contributed substantially to the protocol.; e Britta Hahn** Dentist, Department of Conservative Dentistry and Periodontology, Center of Dental Traumatology, University Hospital Würzburg, Würzburg, Germany. Developed hypothesis, study design, and proofread the manuscript. **both authors contributed equally to the article

**Keywords:** clinical research, clinical study, fiber-reinforced resin restorations, fibre-reinforced composite cantilever fixed partial, glass fiber-reinforced composite resin

## Abstract

**Purpose:**

The goal of this study was to determine the survival rate, success rate, and periodontal health quality (PHQ) of glass fiber-reinforced composite (FRC) resin-bonded fixed dental prostheses (RBFDPs) over an observation period of up to 22 years.

**Materials and Methods:**

RBFDPs reinforced with unidirectional glass fiber strands were fabricated directly (n = 16) or indirectly (n = 31; *n = 1 missing data) for 48 adult patients (24 female, 24 male) and used to replace 39 anterior teeth and 9 posterior teeth in the maxilla (n = 33) or mandible (n = 15) using different framework designs: single-retainer (n = 8), double-retainer (n = 33), or multi-retainer (n = 7). Their fate was classified as a success, functional survival, or failure based on observed complications. PHQ was evaluated by measuring PPD, CAL, SBI, and PI around the abutment teeth and unrestored reference teeth (controls). The clinical quality of the restorations was evaluated according to the Fédération Dentaire Internationale (FDI) criteria. Statistical analysis included Kaplan–Meier survival analysis, Wilcoxon tests, Mann–Whitney U tests, and Chi-squared tests. The significance level was set at P <0.05 for all statistical tests.

**Results:**

The outcome was “failure” in 37 cases (52.9%), “functional survival” (at a mean follow-up of 9 years) in 15 cases (25.7%), and “success” in 18 cases (25.7%). The median time to first complication was 32.0 months. The most common complications were debonding and fracture. Multi-retainer FRC-RBFDPs had the highest complication rates. Most FRC-RBFDPs were of good clinical quality according to FDI criteria. PHQ scores showed significantly higher clinical attachment level (CAL) (pw = 0.027) around abutments (3.40 ± 1.42/controls: 3.09 ± 1.11) and significantly greater plaque accumulation around abutments (pw = 0.008, 3.28 ± 1.03/controls: 2.83 ± 1.02) and pontics (pw = 0.001, 3.50 ± 0.99/controls: 2.83 ± 1.02) than around control teeth. Pocket probing depth (PPD) values were lower for abutments (2.43 ± 0.49) than for control teeth (2.52 ± 0.55). The opposite was true for sulcus bleeding index (SBI) scores (abutments: 0.64 ± 0.76/controls: 0.42 ± 0.71). There were no significant differences in periodontal health variables between younger and older restorations. In group comparisons, mean and median PPD, CAL, and plaque index (PI) scores for abutment and control teeth were higher in older patients than in younger patients.

**Conclusion:**

Based on our survival and complication data, FRC-RBFDPs can be considered short- to medium-term restorations. A 5-year survival rate of approximately 80% can be expected. The most common complications were reparable defects. Thus, repairs can prolong the survival time in many cases. Patient age had a greater effect on PHQ than restoration age. For example, FRC-RBFDPs in older patients were associated with higher levels of plaque accumulation and gingival inflammation around abutments, suggesting that older patients have greater difficulty cleaning the resin-bonded bridge area.

Increasing esthetic expectations and efforts to preserve teeth in patients of all ages have led to growing interest in tooth-colored fixed dental prostheses (FDPs).^35^ Resin-bonded fixed dental prostheses (RBFDPs) are a minimally invasive, cost-effective treatment option for replacing missing teeth in the anterior or posterior region. Fabrication techniques for metal-ceramic FDPs have been optimized and improved many times over the past decades. Accordingly, a large long-term study published in 2014 showed that single-retainer cantilever RBFDPs with retentive abutment tooth preparation achieved a 5-year survival rate of 97%, a 10-year survival rate of 91%, and a 15-year survival rate of 84%.^9^ However, gray discoloration and loss of translucency in situ can compromise the esthetics of the bridge abutment teeth.^12,41^

Fiber-reinforced composite (FRC) offers an esthetic, metal-free alternative to metal-ceramic in resin-bonded bridge fabrication. This material was first investigated in the 1980s and 1990s.^20^ Today, fiber-reinforced composites are used not only for RBFDPs, but also for posts, splints, retainers, and removable dentures.^13,18,23,36,49,50,59^ Case reports describe the use of FRC with unidirectional glass fiber strands, for example, to fabricate splints to stabilize periodontally compromised loose teeth. Reparability is often emphasized as an advantage of using FRC to fabricate splints. However, varying degrees of tooth mobility can compromise the longevity of these restorations. In this context, debonding is a known complication.^37,43^

Comprehensive reports of the long-term longevity and quality of FRC-RBFDPs are scarce, but studies with up to 15 years of follow-up have shown promising results. Five-year survival rates ranging from 73.5% to 93% have been reported.^47,51,54,56^ Three- to five-year survival rates ranging from 73.4% to 94.5% have been reported in systematic reviews. This wide variation between studies may be due to a lack of standardization of study conditions and small sample sizes.^2,4,10,45,53^ For anterior FRC-RBFDPs alone, 3- and 5-year survival rates ranging from 64% to 97.7% have been reported.^3,17,28,55^ For posterior FRC-RBFDPs, Barabanti et al^6^ reported a 15-year mean survival rate of 89.2%. Other investigators reported a cumulative survival rate of 93% for direct FRC-RBFDPs after a mean follow-up of 4.4 years.^33^ Survival rates also varied significantly depending on the retention mechanism used. Inlay-retained FRC-RBFDPs were significantly more likely to survive than hybrid-anchored FRC-RBFDPs at a mean follow-up of 4.9 years.^19^

In addition to the general advantages of resin-bonded bridges, fiber reinforcements are added to improve the mechanical properties of composite resins and their tooth-like wear resistance while maintaining the natural translucency of the restoration.^14^ Because fiber-reinforced composite resins have a lower modulus of elasticity than non-precious metals or ceramics, they are also believed to reduce stresses in the bond zone, thereby reducing restoration failure and complication rates.^51^ Other advantages include improved adhesion of composite resin luting materials to the framework, superior esthetics, and greater physiological stiffness. FRC bridges can be repaired by secondary addition of composite resin or fiber strands.^54,55^ In the case of direct FRC-RBFDPs, gaps can be restored in a single session.^33^ Compared with ceramics, composite resins have been described as having less favorable surface properties associated with increased plaque accumulation, which is thought to increase the risk of periodontal complications.^7^ In FRC-RBFDP design, the framework is fabricated directly, indirectly, or semi-directly using unidirectional prepregs (composites pre-impregnated with glass fiber bundles) embedded in a PMMA/bis-GMA matrix^2,39^ and then veneered with a moldable composite resin.

The aim of the present study was to determine the long-term survival, success rate, and periodontal health impact of glass fiber-reinforced composite resin-bonded bridges after a mean follow-up of 9 years.

## MATERIALS AND METHODS

This study is based on data from follow-up examinations of FRC-RBFDPs used to replace missing teeth in patients at the outpatient clinic of the Department of Conservative Dentistry and Periodontology, University Hospital Würzburg, from 2000 to 2015. In addition, 61 FRC-RBFDP restorations were clinically evaluated in a previous pilot study conducted in 2018 to test the feasibility of the present study. Twenty-nine of the aforementioned FRC-FDPs in 23 patients were then clinically re-evaluated from June to September 2023. In addition, a medical record search yielded relevant data on an additional 31 FRC-RBFDPs in 25 patients.

The project was pre-approved by the University of Würzburg Ethics Committee (approval number: 136/22) on October 18, 2022. It is designed as a retrospective, observational study and has been registered in the German Clinical Trials Register (DRKS; ID-Nr: DRKS00036333).

Inclusion Criteria: Anterior or posterior FRC-RBFDPs bonded from 1/1/2000 to 12/31/2015; patients who were of legal age (≥18 years) at the time of recall, had the capacity to give informed consent, signed the consent form, and were male, female, or non-binary gender.

Exclusion Criteria: immunosuppressive treatment, radiotherapy to the head and neck; pregnancy or breastfeeding; use of antibiotics within the last 28 days; coagulation disorders or anticoagulation therapy; participation in another clinical study within the last 30 days; leukemia, cirrhosis of the liver, sarcoidosis, active hepatitis, tuberculosis and/or AIDS; poor oral health (severe periodontal disease, multiple advanced carious lesions, massive calculus); any disease associated with impaired oral hygiene (eg, Parkinson’s disease, Huntington’s disease, dementia); dental professionals and dental students.

All indirect FRC-RBFDPs were fabricated by the same dental technician in our in-house laboratory using unidirectional glass fiber strands (everStick C&B, Stick Tech, GC) and resin composites (Enamel plus HFO (Micerium; Avegno, Italy), Biostyle OD2 (Dreve, Unna, Germany), SR Adoro (Ivoclar Vivadent AG, Schaan, Liechtenstein)). The direct technique required a complete intraoral fabrication. Thus, fiber-reinforced composite bundles were adhesively fixed on abutment teeth (in cases involving dual- or multi-retainer designs, connecting all abutment teeth). The pontics were subsequently incrementally shaped using the same materials as mentioned above. In five cases, the patients’ own natural tooth was used as a pontic.

All study restorations were placed by trained conservative dentists. If there was sufficient overjet (>0.5 mm), the FRC-RBFDPs were cemented without preparation. If there was insufficient overjet (<0.5 mm), a 0.5 mm layer thickness was used for the retainers. In two cases identified in the medical record search, an FRC-RBFDP was used to replace multiple teeth. FRC-RBFDPs with a single-retainer, dual-retainer, or multi-retainer design were included in the study.

All diagnostic examinations were performed by a calibrated study dentist according to a standardized protocol using a treatment light, basic dental instruments, and a loupe (Zeiss, 3.5× magnification). The following clinical variables were evaluated: tooth mobility according to Lindhe & Nymann (1977), sulcus bleeding index (SBI) (Mühlemann & Son 1971), pocket probing depth (PPD), clinical attachment level (CAL), bleeding on probing (BoP), periodontal screening index (PSI), and plaque index (PI) (Turesky).^46^ The FDI (Fédération Dentaire Internationale) criteria were used to evaluate the clinical quality and performance of the restorations over time.^22^

**Fig 1a to c:**
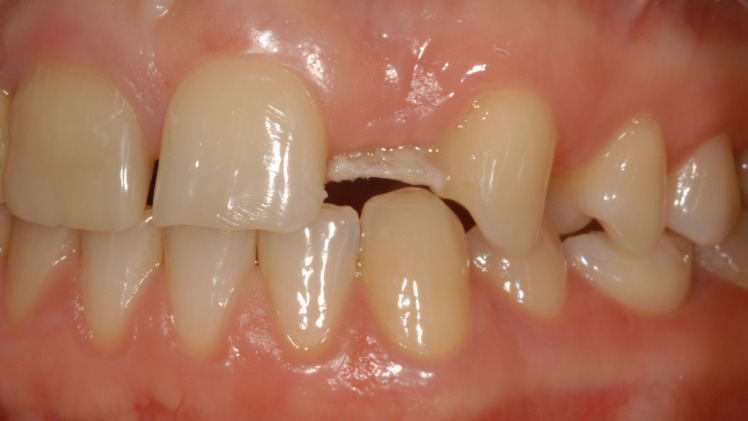

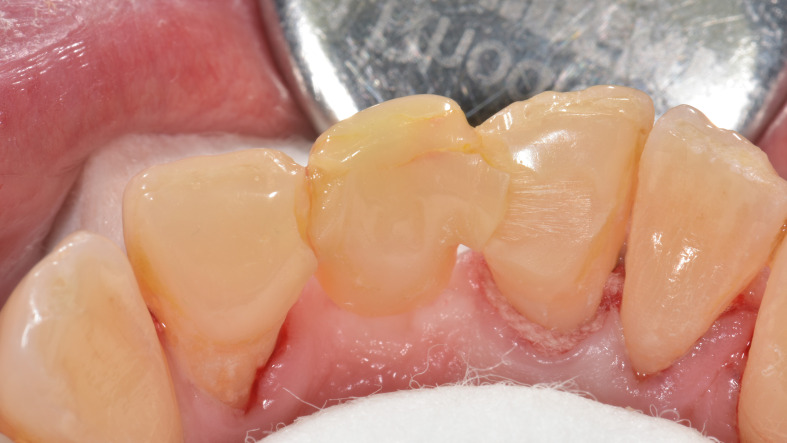

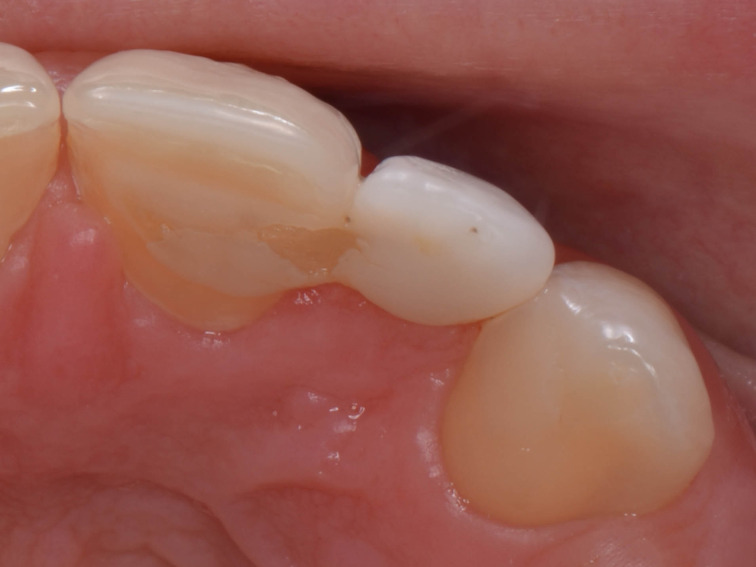
Clinical examples of “failure” (a), “functional survival” (b), and “success” (c).

Restorations that were still in place and fully intact at follow-up were classified as success (S). Those in place but defective at follow-up or requiring repair due to complications during their service life were classified as functional (“functional survival,” FS). Restorations that were lost or irreparably damaged at follow-up were classified as failed (“failure,” F) (Fig 1).

Statistical analysis was performed using SPSS Statistics (version 29.0, IBM). Graphs were generated with GraphPad Prism (version 5.01, GraphPad Software). To avoid confounding, only one FRC-RBFDP per patient was included in the statistical analysis. For patients with multiple restorations, one FRC-RBRDP was randomly selected and included in the analysis. As this was an observational study, a descriptive analysis of all P values was appropriate. Due to the limited patient pool, patient selection was not randomized. Therefore, the results of this study may be biased and only partially representative of the overall population. The significance level for all statistical tests was set at P <0.05.

For descriptive analysis of individual variables, absolute and relative frequencies were calculated for nominal and ordinal variables. Mean, standard deviation, median, minimum, maximum, and 25th and 75th percentiles were used for metric variables. Functional survival and success were analyzed separately using the Kaplan–Meier method.^25^

Wilcoxon tests were used to compare scores for the periodontal health assessment variables PPD, CAL, PI, and SBI between abutments and unrestored reference teeth (controls). Mann–Whitney U tests were used to test for differences in PPD, CAL, PI, and SBI scores between the 25% youngest and 25% oldest patients and between the 25% youngest and 25% oldest FRC-RBFDPs. Box plots were generated to graphically represent the data. The chi-square test of independence was used to test for correlations between two or more categorical variables. For ordinal data, Spearman’s rank correlation coefficient was used to measure the strength of association between two variables. The statistical power of correlation coefficients (ρ) was interpreted according to Cohen’s method.^11^

## RESULTS

### General Data

The evaluated FRC-RBFDPs (n=48) had a mean age of 9.64 ± 6.1 years (median: 9.88 years). Kaplan–Meier curves were calculated using data from 70 patients. The mean follow-up time was 9.00 ± 5.49 years, and the median was 9.25 years. Further information regarding the FRC-RBFDPs’ age is presented in Table 2. General data is shown in Table 1.

**Table 2:** Descriptive analysis of FRC-RBFDP age (in years) by placement date

	N	Mean	SD	Median	Min	Max
Evaluated RBFDPs	48	9.64	6.08	9.88	0	22.58
Kaplan–Meier	70	9.00	5.49	9.25	0	22.58


**Table 1:** General data

	Characteristic	Value
Study population	Number of patients	48
Gender	50% female, 50% male
Patient age (years)Mean ± SD (median)	48.58 ± 19.75 (42.0)
Bridge location	Mandible	68.8%
Maxilla	31.3%
Anterior	81.3%
Posterior	18.8%
Bridge design	Single-retainer	16.7%
Double-retainer	68.8%
Multi-retainer (>2)	14.6%
Fabrication technique	Direct	33.3%
Indirect	64.6%
Not specified	2.1%
Cause of tooth loss	Traumatic	16.7%
Congenital	18.8%
Other causes	31.3%
Periodontal disease	20.8%
Not specified	12.5%


#### Survival and failure analysis

**Fig 2:**
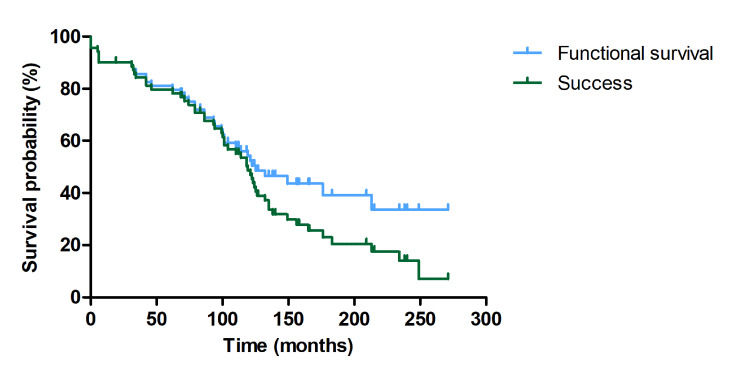
Kaplan–Meier survival probability curves for functional survival and success.

The fate of the evaluated FRC-RBFDPs was classified as “failure” in 52.9% of cases (n = 37), “functional survival” in 21.4% (n = 15), and “success” in 25.7% (n = 18). Kaplan–Meier survival probability curves for “functional survival” and “success” are shown in Figure 2. Annual failure rates are presented in Table 3. The median time to failure was 10.42 years (95% CI: 7.47 to 13.36), and the median time to success was 9.92 years (95% CI: 8.76 to 11.08).

**Table 3:** Annual failure rates

	Failure rates considering previous losses [%]	Lost restorations (absolute numbers)	Remaining restorations (absolute numbers)
Baseline	–	–	70
1st year	10	7	63
2nd year	0	0	63
3rd year	4.76	3	60
4th year	5	3	57
5th – 10th year	24.56	14	43
10th – 15th year	18.60	8	35
15th – 20th year	5.71	2	33
20th – 23rd year	0	0	33


The median time to failure was 12.42 years (95% CI: 8.49 to 16.34) for FRC-RBFDPs with one retainer, 10.16 years (95% CI: 7.89 to 12.44) for those with two retainers, and 7.16 years for those with >2 retainers. There was no significant difference in survival between the three design groups.

The median time to failure for maxillary FRC-RBFDPs was 10.08 years (95% CI: 7.57 to 12.60). The median time to failure for mandibular FRC-RBFDPs could not be determined due to an insufficient number of events (n = 8).

The median time to failure was 9.92 years (95% CI: 7.75 to 12.08) for anterior restorations and 14.6 years (95% CI: 8.1 to 21.23) for posterior restorations. The difference was not statistically significant.

A total of 48 FRC-RBFDPs were included in the descriptive analysis of complications. Five different types of complications were distinguished: chipping, fracture, debonding, delamination, and removal of one retainer. 14.6% of the FRC-RBFDPs were complication-free, while 85.4% experienced at least one complication. The relative and absolute frequencies of the five types of complications are shown in Table 4.

**Table 4:** Descriptive analysis of complications

	Chipping	Fracture	Debonding	Delamination	Removal of one retainer
%	N	%	N	%	N	%	N	%	N
At least one event	14.6	7	33.3	16	39.6	19	4.2	2	6.3	3
Multiple events	4.2	2	4.2	2	16.7	8	2.1	1	0	0


The median time to first complication was 32.0 months (95% CI: 9.96 to 54.04). The complication rates for each adhesive bridge design group are shown in Table 5. There were significant differences in complication rates between design types (single-retainer, double-retainer, and multi-retainer): χ^2^(2) = 7.84, pcq = 0.02. Paired comparisons of the three groups showed that multi-retainer FRC-RBFDPs had increased complication rates compared to the other two groups, and the difference was significant, especially when compared to single-retainer FRC-RBFDPs (pcq = 0.026).

**Table 5:** Number and percentage of complications with FRC-RBFDPs by retainer design

	Number of complications	Total
≤ 2	>2
Design	One-retainer	Number	8	0	8
	% of group	100.0%	0.0%	100.0%
Two-retainer	Number	27	6	33
	% of group	81.8%	18.2%	100.0%
Multi-retainer	Number	3	4	7
	% of group	42.9%	57.1%	100.0%
Total sample %	Number	38	10	48
79.2%	20.8%	100.0%	


The strength of evidence was insufficient to confirm an association between any of the three design types or any of the five complication types (pcq >0.05). However, there was a trend toward a higher frequency of debonding events in FRC-RBFDPs with two or more retainers than in those with only one retainer.

Similarly, no association was found between the number of complications and the location of the FRC-RBFDP in the arch or jaw (anterior vs posterior, maxilla vs mandible), nicotine use, or periodontitis (pcq > 0.05).

### Clinical Data Analysis

#### Clinical quality

The clinical quality scores for the restorations evaluated according to the FDI criteria are shown in Table 6. Twenty-three FRC-RBFDPs were included in the analysis. In most cases, the restorations were of good clinical quality.

**Table 6:** Distribution of clinical quality scores for restorations assessed according to the FDI criteria

Score	%	n	%	n	%	n	%	n	%	n	%	n
Surface luster	Marginal discoloration	Color stability, translucency	Anatomic form, proximal contact	Fracture, retention	Marginal adaptation
	Wear	Patientʼs view	Cleanability	Postoperative hypersensitivity	Caries, erosion, abfraction	Periodontal response
1	73.9	17	21.7	5	52.2	12	56.5	13	26.1	6	26.1	6
2	21.7	5	56.5	13	478	11	26.1	6	13.0	3	47.8	11
3	4.3	1	21.7	5			17.4	4	52.2	12	21.7	5
4									8.7	2	4.3	1
1	13.0	3	47.8	11	26.1	6	100.0	23	82.6	19	30.4	7
2	34.8	8	43.5	10	60.9	14			8.7	2	39.1	9
3	47.8	11	4.3	1	13.0	3			4.3	1	26.1	6
4	4.3	1	4.3	1					4.3	1	4.3	1


The data were also analyzed to determine if there was an association between smoking and restoration quality. Of the 23 patients included in the study, 7 were smokers and 16 were non-smokers. A moderate correlation was found between smoking and lower quality scores in the categories of “surface luster” (Spearman’s ρ = 0.483, p_s = 0.019_) and “periodontal response” (Spearman’s ρ = 0.452, p_s_ = 0.031). The relative and absolute distributions of FDI scores in smokers and non-smokers are shown in Table 7.

**Table 7:** Restoration quality scores in smokers versus non-smokers according to the FDI criteria

Score	Surface luster	Periodontal response
Non-smokers	Smokers	Non-smokers	Smokers
%	n	%	n	%	n	%	n
1	87.5	14	42.9	3	43.8	7	0.0	0
2	12.5	2	42.9	3	37.5	6	42.9	3
3	0.0	0	14.3	1	12.5	2	57.1	4
4	n.s.		n.s.		6.3	1	0.0	0


#### Periodontal health quality (PHQ)

**Fig 3a to d:**
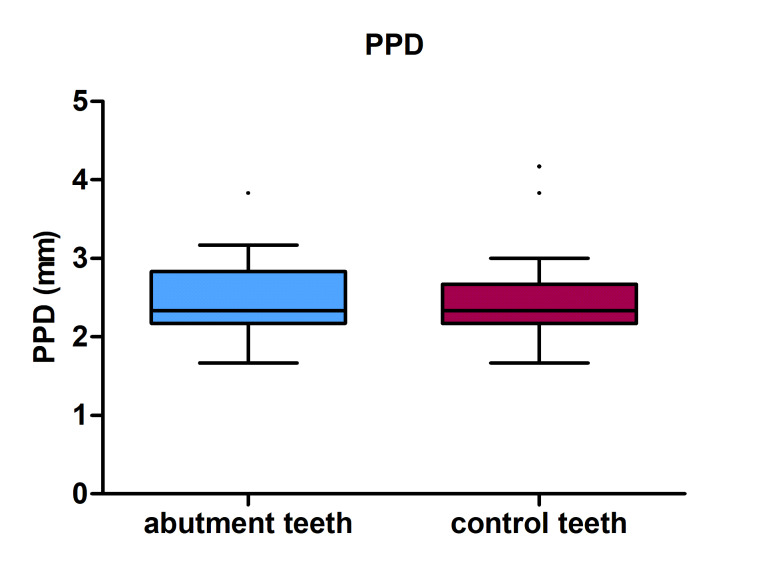

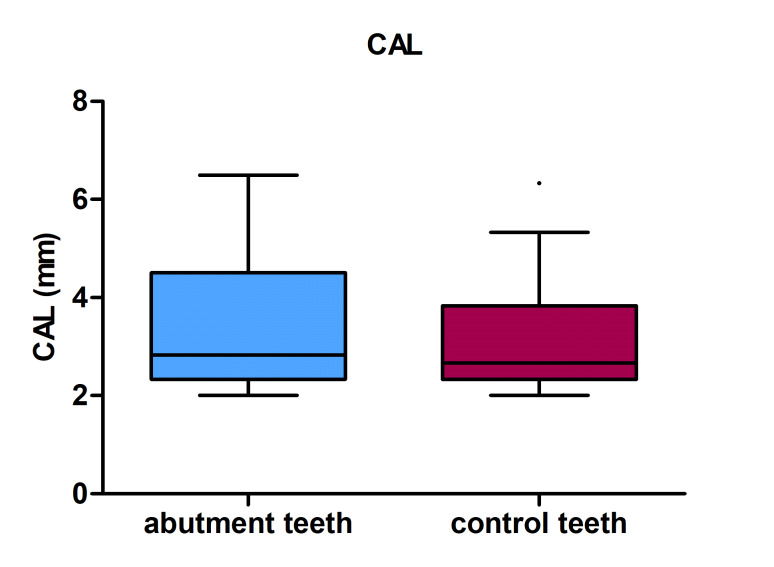

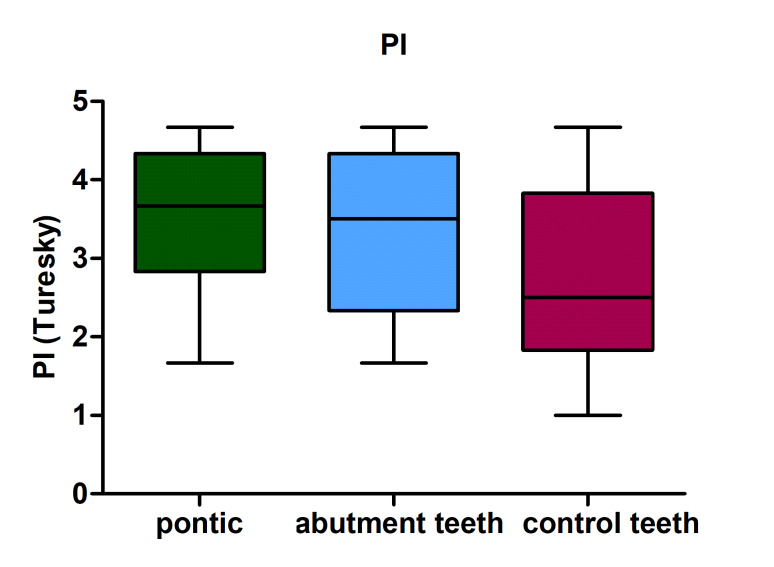

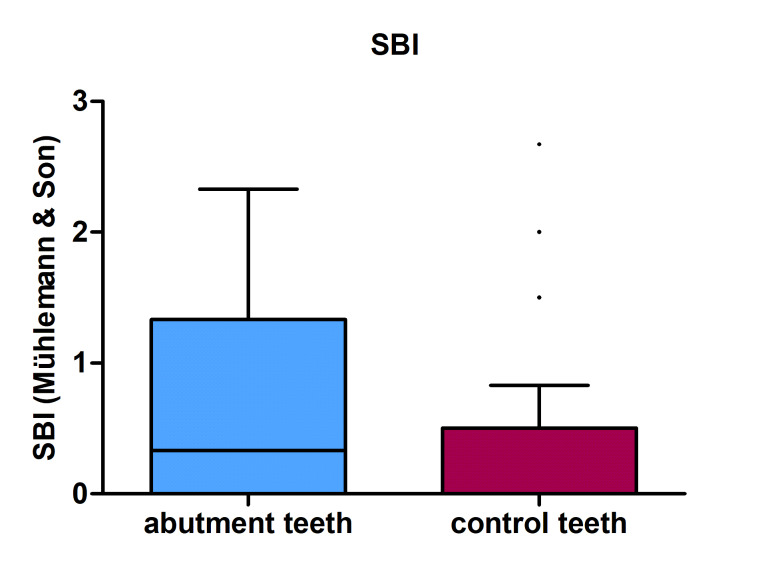
PPD, CAL, PI, SBI in abutment teeth and control teeth.

Descriptive statistics for the PHQ variables PPD, CAL, PI, and SBI in abutments, control teeth, and pontics are shown in Table 8 and Figure 3. All abutments had higher mean and median CAL, PI, and SBI scores than control teeth. Pontics had higher mean and median plaque accumulation scores than abutment and control teeth. PPD was the only variable with lower mean values for abutments than for control teeth. Significant differences were observed in CAL values (pw = 0.027) and PI scores between abutments and controls (pw = 0.008) and between pontics and control teeth (pw = 0.001).

**Table 8:** Descriptive statistics for PPD, CAL, PI, and SBI

		Abutments	Control teeth	Pontics
PPD	Mean ± SD	2.43 ± 0.49	2.52 ± 0.55	n.s.
Median (Q1, Q3)	2.33 (2.17, 2.83)	2.33 (2.17, 2.67)
Minimum	1.67	1.67
Maximum	3.83	4.17
CAL	Mean ± SD	3.40 ± 1.42	3.09 ± 1.11	n.s.
Median (Q1, Q3)	2.83 (2.33, 4.50)	2.67 (2.33, 3.83)
Minimum	2.00	2.00
Maximum	6.50	6.33
PI	Mean ± SD	3.28 ± 1.03	2.83 ± 1.02	3.50 ± 0.99
Median (Q1, Q3)	3.50 (2.33, 4.33)	2.50 (1.83, 3.83)	3.67 (2.83, 4.33)
Minimum	1.67	1.00	1.67
Maximum	4.67	4.67	4.67
SBI	Mean ± SD	0.64 ± 0.76	0.42 ± 0.71	n.s.
Median (Q1, Q3)	0.33 (0.00, 1.33)	0.00 (0.00, 0.50)
Minimum	0.00	0.00
Maximum	2.33	2.67


#### Periodontal health quality vs patient age

To investigate the effect of patient age on periodontal health quality, the scores for the above PHQ variables were compared within and between two age groups containing the youngest 25% and the oldest 25% of patients, respectively. No significant differences in PPD and CAL were observed between abutment and control teeth within either of the two age groups (pw >0.05). Close proximity between mean PPD and CAL levels observed between the two age groups was generally. However, the mean PPD values for the older patients and the mean PPD and CAL values for the younger patients were higher around the control teeth. The only exception was the mean CAL around the abutment teeth in the oldest patients, which was higher than the mean CAL around the control teeth in the same age group.

**Fig 4a to d:**
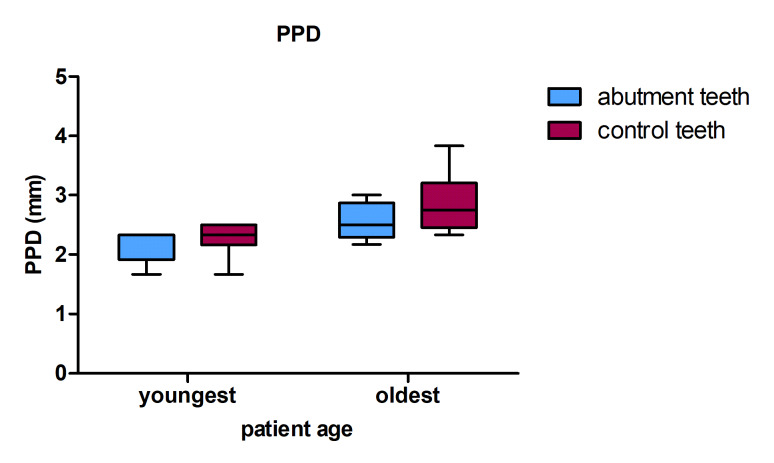

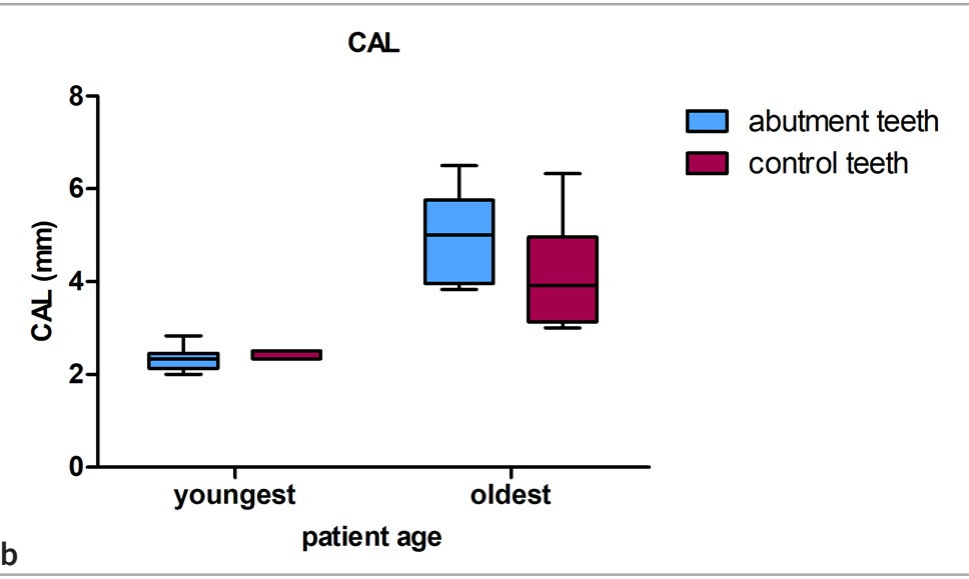

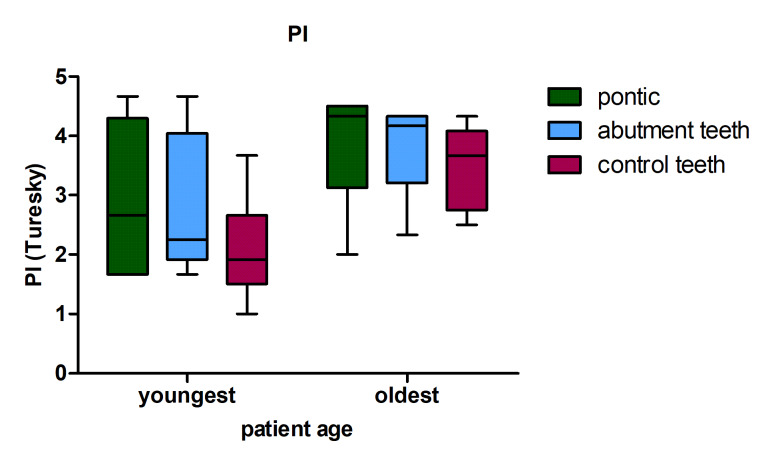

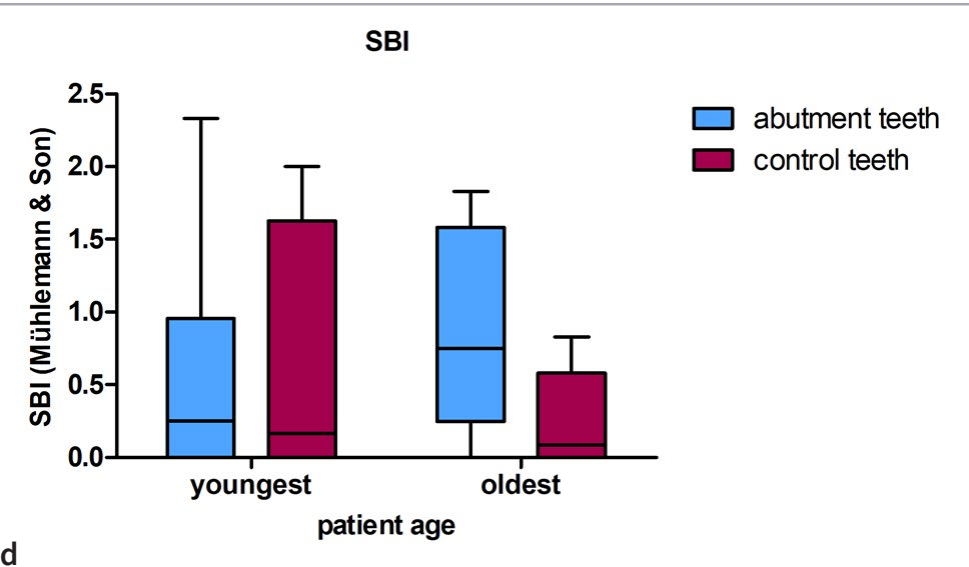
PPD, CAL, PI, and SBI versus patient age.

Group comparisons of the oldest and youngest 25% of patients revealed significant differences in CAL around abutment teeth (pu = 0.004) and significant differences in CAL (pu = 0.003), PPD (pu = 0.027), and PI (pu = 0.016) around control teeth. For both abutments and controls, the mean and median PPD, CAL, and PI scores were higher in the oldest than in the youngest 25% of patients. For abutments, the mean and median SBI scores were higher in the older group compared with the younger group. The opposite was true for control teeth, where the corresponding mean and median were higher in the youngest than in the oldest 25% of patients. For pontics, the mean and median plaque accumulation levels were higher in the older comparison group. The results are shown in Figure 4.

#### Periodontal health quality vs restoration age

As in the above analysis, the FRC-RBFDPs were also examined for the effects of increasing restoration age on the specified periodontal health variables, plaque accumulation, and severity of gingival inflammation. There were no significant differences between younger and older FRC-RBFDPs for any of the variables studied. This was true for both abutments and controls. For abutment and control teeth, close proximity between the mean and median values for older and younger restorations was generally observed.

## DISCUSSION

Of the 70 FRC-RBFDPs evaluated, 47.1% showed survival at a mean follow-up of 9 years. The survival rates observed in this relatively long-term observational study are consistent with similar studies that have reported 2–5 year survival rates ranging from 64% to 97.7%. The significant variation between studies may be due to a lack of standardization of study conditions and small sample sizes of FRC-RBFDPs.^2,4,17,28,45,47,51,53–56^

Tooth-related factors such as arch (maxilla or mandible) and tooth type (anterior or posterior) had no significant effect on FRC-RBFDP survival. The observed trend toward shorter survival of anterior FRC-RBFDPs restorations may be due to the effects of divergent shear forces, which have a greater propensity to cause debonding, whereas posterior teeth and restorations are subject to masticatory forces with axial effects. This assumption is consistent with reports of superior survival rates for posterior FRC-RBFDPs compared to anterior FRC-RBFDPs.^6,17,56^ Similarly promising survival data have been reported for all-ceramic zirconia RBFDPs of various designs in the canine and posterior region.^1,58^

Various studies of metal-ceramic and all-ceramic RBFDPs have clearly demonstrated the superiority of single-retainer RBFDPs placed in the anterior region.^8,27,42,45^ Given their excellent long-term performance, single-retainer all-ceramic zirconia RBFDPs are now considered an established treatment option.^26,27,40–42^ Sasse and Kern^42^ hypothesize that RBFDPs with a two-retainer design tend to have lower survival rates because the abutment tooth movement that occurs during functional loading, although minimal, acts in different directions. Torquing and shear forces act in different directions and may therefore result in debonding. Although this effect cannot be completely avoided when the FRC-RBFDP is designed with two or more retainers, only a trend toward shorter survival of FRC-RBFDPs with these retainer designs was observed in the present study. The non-significance of this finding must be interpreted in the context of high loss rates and inhomogeneous group sizes. For the majority of lost RBFDPs, it is not possible to determine retrospectively whether failure was due to design disadvantages or other factors. However, given significant differences in complication rates between designs, the use of single-retainer FRC-RBFDPs in the anterior region may be preferred.

In addition to these high loss rates, fracturing and debonding are common complications with resin-bonded bridges. The median time to first complication was 32.0 months. In most cases, the first complication was a repairable defect. RBFDPs with multi-retainer designs have been shown to be particularly prone to complications. As has been confirmed for resin-bonded bridges made from other materials, FRC-RBFDPs should not be fabricated using a multi-retainer design.^8,42^ Data suggest that it may be possible to improve the mechanical properties of these bridges by modifying the design and placing an additional fiber bundle in the area of the pontic.^57^ The high rate of restorations with repeated debonding suggests that the old composite-cement residue was not completely removed prior to rebonding. Although these complications can be repaired, they are disruptive events that result in additional treatment time and cost for clinicians and patients. The extent to which this problem affects patient satisfaction cannot be determined from the results of the present study because of the high loss rate and the inability of affected patients to provide useful information. Therefore, the high level of patient satisfaction in the present study is not representative.

The mechanical properties of adhesive bridges are influenced by the choice of fabrication material, design, and technique. FRC-RBFDPs with high-volume substructures have been shown to have significantly better survival than those with low-volume substructures^16^. In addition, the design and location of the glass fiber strands appear to have a significant effect on their fracture resistance.^44^ Another study suggests that the effect of reinforcement is not significant unless the fibers are located on the tension side, with the fiber direction perpendicular to the direction of force.^15^ Therefore, it may be possible to mitigate failures in the pontic region by using a cross-sectional design and increasing the pontic layer thickness.^32^ Biological and mechanical factors in the oral cavity also influence the aging of restorative materials and have a corresponding effect on the longevity of FRC-RBFDPs. Vallittu^48^ observed irreversible damage due to hydrolytic degradation of the siloxane bonds formed between the glass fibers and the polymer matrix during long-term storage in water. As a result, the affected restorations had lower flexural strength and elasticity modulus values. The results of these material property studies suggest that the survival time of FRC-RBFDPs is limited, regardless of design and fabrication technique.

Most of the FRC-RBFDPs that were still present and clinically evaluated at follow-up were of acceptable clinical quality. Due to their material properties, they showed signs of wear, which is a significant drawback of these restorations. The increased complication rates may be due to structural weakening of the FRC-RBFDPs. As in previous studies, composite esthetics and periodontal response scores at the restoration sites were significantly lower in smokers.^24,29,30,38^

The present study of FRC-RBFDPs with a mean age of 9.6 years showed no evidence of a negative effect of restoration age on PHQ. The increased attachment loss observed around the abutments was likely due to physiologic ridge atrophy.^5^ As in other studies of composite restorations,^31,34^ increased plaque accumulation occurred around pontics and abutments. The age of the restorations appears to play a minor role. FRC-RBFPD age had no effect on PHQ or gingivitis severity. However, older patient age, presumably associated with declining oral hygiene quality, is a factor in increased biofilm accumulation. In addition, the majority of FRC-RBFDPs evaluated in this study had a two- or multi-retainer design. These retainer designs are known to be associated with poorer oral hygiene performance than a single-retainer design.^21^ Although this effect was observed in all patients, it may be easier for younger patients to perform specific oral hygiene measures in the area of the restoration. Increased attachment loss in older patients is a natural consequence of the aging process.

As suggested by other authors, our results confirm that FRC-RBFDPs are an appropriate option for short- to medium-term replacement of missing teeth.^33,52^ They are a cost-effective, minimally invasive, repairable fixed prosthetic treatment option that allows for a dynamic treatment approach.^52^ However, all-ceramic RBFDPs are preferred for long-term tooth replacement due to their better survival and complication rates and highly esthetic appearance.^4,26,27,40–42^

## CONCLUSION

Based on the results of this study, FRC-RBFDPs appear to be a suitable option for short- to medium-term temporization with a treatment horizon of up to 5 years. The expected 5-year survival and success rates for these restorations are approximately 80% and 78%, respectively.

### Acknowledgments

The authors thank Mr. Halbleib for fabricating the indirect FRC-RBFDPs. This study was conducted without any third-party funding.




